# GOing Forward With the Cardiac Conduction System Using Gene Ontology

**DOI:** 10.3389/fgene.2022.802393

**Published:** 2022-03-02

**Authors:** Kan Yan Chloe Li, Andrew C Cook, Ruth C Lovering

**Affiliations:** ^1^ Department of Preclinical and Fundamental Science, Institute of Cardiovascular Science, Functional Gene Annotation, University College London, London, United Kingdom; ^2^ Department of Children’s Cardiovascular Disease, Centre for Morphology and Structural Heart Disease, Institute of Cardiovascular Science, University College London, London, United Kingdom

**Keywords:** gene ontology (GO), cardiac conduction, heart development, biocuration, annotation

## Abstract

The cardiac conduction system (CCS) comprises critical components responsible for the initiation, propagation, and coordination of the action potential. Aberrant CCS development can cause conduction abnormalities, including sick sinus syndrome, accessory pathways, and atrioventricular and bundle branch blocks. Gene Ontology (GO; http://geneontology.org/) is an invaluable global bioinformatics resource which provides structured, computable knowledge describing the functions of gene products. Many gene products are known to be involved in CCS development; however, this information is not comprehensively captured by GO. To address the needs of the heart development research community, this study aimed to describe the specific roles of proteins reported in the literature to be involved with CCS development and/or function. 14 proteins were prioritized for GO annotation which led to the curation of 15 peer-reviewed primary experimental articles using carefully selected GO terms. 152 descriptive GO annotations, including those describing sinoatrial node and atrioventricular node development were created and submitted to the GO Consortium database. A functional enrichment analysis of 35 key CCS development proteins confirmed that this work has improved the *in-silico* interpretation of this CCS dataset. This work may improve future investigations of the CCS with application of high-throughput methods such as genome-wide association studies analysis, proteomics, and transcriptomics.

## 1 Introduction

The electrical cardiac conduction system (CCS) consists of the sinoatrial node (SAN), atrioventricular node (AVN) and the ventricular conduction system (VCS). The VCS can be divided into the following discrete components: bundle of His (or atrioventricular bundle (AVB)), which bifurcates into the left bundle branch (LBB) and right bundle branch (RBB) and the distal Purkinje fiber network (PFN), as reviewed in detail by Kennedy et al. (2016) (Kennedy et al., 2016). These components are essential for the coordinated contraction of the cardiac chambers. The SAN is a slow-conducting node formed in the sinus venosus and serves as the primary pacemaker of the heart. The normal cardiac action potential (AP) is initiated in the SAN, then rapidly propagates through the atrial myocardium, inducing atrial contraction. The AP is delayed once it arrives at the slow-conducting AVN which enables the atria to fully contract before ventricular contraction begins (van Weerd and Christoffels 2016). The delay at the AVN is important for complete filling of the ventricles before ventricular contraction. The AP propagates from the AVN to the sheathed and fast-conducting AVB. As the AVB runs through the central fibrous body onto the crest of the ventricular septum, it provides the only electrical route from atrial to ventricular myocardium in normal hearts (van Weerd and Christoffels 2016). From the AVB, the AP is conducted to the LBB and RBB, ultimately terminating at the PFN and activating the ventricular myocardium. Dysregulation of the development or function of any one of the CCS components can cause conduction abnormalities and, in some cases, early death (Wolf and Berul 2006).

A network of essential transcription factors and signaling pathways are involved in CCS development through the suppression of the working myocardial gene program and stimulation of pacemaker-like properties (van Weerd and Christoffels 2016; van Eif et al., 2018). For example, short stature homeobox 2 (SHOX2) and bone morphogenic protein 4 (BMP4) are known to be important for SAN formation (Hoogaars et al., 2007; Mommersteeg et al., 2007; Wiese et al., 2009; Puskaric et al., 2010; Tessadori et al., 2012; Liang et al., 2015; van Weerd and Christoffels 2016). Furthermore, in mouse, AVC-restricted deficiency of bone morphogenetic protein receptor type 1A (Bmpr1a, alias Alk3) has been shown to cause defective AVN morphogenesis (Gaussin et al., 2002; Stroud et al., 2007). As the speed of the electrical impulse is determined by the functionality of the gap junctions and ion channels, precise regulation of these proteins is required for CCS function and appropriate regulation of heart contractions. For example, the gap junction protein alpha 1 (GJA1), gap junction protein alpha 5 (GJA5) and sodium voltage-gated channel alpha subunit 5 (SCN5A) are essential for rapid conduction between atrial cardiomyocytes (Marionneau et al., 2005; Dobrzynski et al., 2007), consequently, these are expressed at a low level in the slow-conducting SAN. In contrast, the expression of low-conductance junctional channels, gap junction protein delta 3 (GJD3) and gap junction protein gamma 1 (GJC1), in the SAN ensure slow conduction of the electrical impulse in this node (Kreuzberg et al., 2005; Gros et al., 2010; van Eif et al., 2019). Several T-box transcription factors (TBXs) regulate gene expression in the CCS. TBX3 is expressed in the CCS, including the SAN, and is crucial for repressing atrial differentiation (Wiese et al., 2009). Murine Tbx3 deficiency results in aberrant development of the conduction system, whereas overexpression leads to formation of ectopic pacemakers (Hoogaars 2004; Bakker et al., 2008). Whereas, Tbx5, Nkx2-5 (NK2 homeobox 5) and Id2 (inhibitor of DNA binding 2) are essential for AVB and bundle branch development (Moskowitz et al., 2004; van Weerd and Christoffels 2016). For example, Nkx2-5 haploinsufficiency in mice causes severely hypoplastic Purkinje fibers. In addition, human mutations in NKX2-5 are associated with progressive cardiomyopathy and atrioventricular block (Pashmforoush et al., 2004).

Although it is known that CCS development relies on the above and other genes, which are involved in complex regulatory networks, not all of this data is captured in an organized computer accessible manner. This can compromise the analysis of data from high-throughput methodologies, such as genome-wide association studies (GWAS), exome sequencing, transcriptomics, and proteomics. High-throughput analyses are powerful tools that are increasingly being used to investigate cardiovascular diseases, many of which are associated with CCS dysfunction (Glinge et al., 2020). Interpretation of these data relies on access to high-quality descriptions of cellular and physiological roles of genes and proteins. The aim of this study is to provide Gene Ontology (GO) annotations that capture the role of key proteins in CCS development (Supplementary Tables S1, S2). These freely available annotations will support an improved interpretation of cardiac-relevant high-throughput analyses and are accessible via the major gold-standard biological knowledgebases (Ashburner et al., 2000; The Gene Ontology Consortium 2019).

The Gene Ontology Consortium provides two publicly available resources: a structured, controlled vocabulary, the Gene Ontology (http://geneontology.org/) (Ashburner et al., 2000) and a database of biological knowledge regarding the functions of genes and gene products in a written and computable form (The Gene Ontology Consortium 2019). GO can describe the characteristics of genes and gene products in any organism at three levels: biological process, cellular component and molecular function (Khodiyar et al., 2011). Following the creation of 152 descriptive GO annotations, we have demonstrated how our contribution to the GO database can improve the investigation of heart disease GWAS and transcriptomic datasets.

## 2 Methods

### 2.1 Gene Ontology Annotation

A list of 35 key proteins known to be required for CCS development was generated based on a review by van Weerd and Christoffels (2016) (van Weerd and Christoffels 2016) (“key CCS development proteins”; Supplementary Tables S1, S2). A list of 14 “priority proteins” was identified from these 35 proteins (BMP2, BMPR1A, GJA5, GJD3, IRX3, ISL1, NKX2-5, PITX2, SHOX2, TBX2, TBX3, TBX5, TBX18 and TBX20) and prioritized for annotation. Articles suitable for curation were identified from van Weerd and Christoffels’ review or PubMed searches using the HUGO Gene Nomenclature Committee (HGNC) approved gene symbol (Tweedie et al., 2021) and CCS-relevant terms.

**FIGURE 1:**
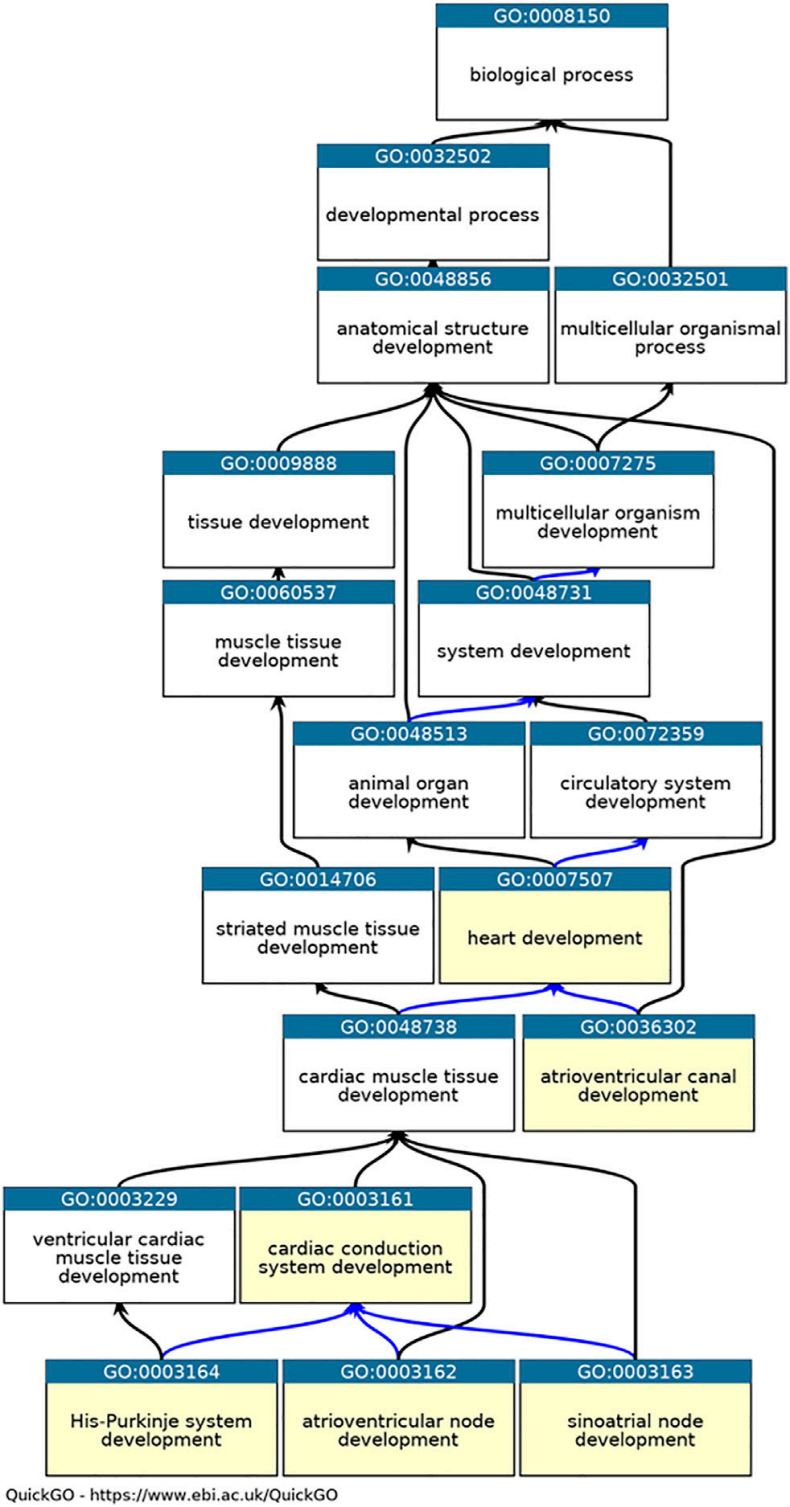
Ontology relevant to cardiac conduction system development. QuickGO graph of the part of the heart development ontology (www.ebi.ac.uk/QuickGO). Six GO terms used as GO slims are highlighted in yellow: GO:0003161 cardiac conduction system development, GO:0003162 atrioventricular node development, GO:0003163 sinoatrial node development, GO:0003164 His-Purkinje system development, GO:0036302 atrioventricular canal development and GO:0007507 heart development. The is_a relations between the GO term are indicated by black arrows, the part_of relations as blue arrows ((Ashburner et al., 2000), (The Gene Ontology Consortium et al., 2021)).

Annotations were created following existing GO Consortium guidelines (Lovering et al., 2018) and included in GO Consortium member databases, annotation files and browsers (The Gene Ontology Consortium 2019). In addition, appropriate annotation extension statements were included to describe the location of the process or function using ontology terms from Uberon (Haendel et al., 2014) (for anatomical structures) and the Cell Ontology (for cell types) (Diehl et al., 2016). In cases where annotations were associated with mouse or zebrafish proteins, the HGNC orthology prediction tool (HCOP) was used to identify the orthologous human protein (Tweedie et al., 2021). Following GO Consortium conventions, the mouse and zebrafish annotations, including annotation extension information, were then copied across to the human ortholog using the evidence code “Inferred from Sequence or structural Similarity” (ISS), the orthologous UniProt identifier was included in the “with” field (Balakrishnan et al., 2013). Six CCS development-relevant GO terms (Figure 1) were used as GO-slims (Mi et al., 2019) to investigate the annotations associated with the 35 key CCS development proteins (annotations downloaded using QuickGO (Binns et al., 2009), 11/05/21).

### 2.2 Functional Enrichment Analysis

GO functional enrichment analysis of our 35 key CCS development proteins (Query Set) was performed on two datasets: October 2020 (start of the project) and March 2021 (end of the project) by using the web-based application, VisuaL Annotation Display (VLAD): (http://proto.informatics.jax.org/prototypes/vlad/) (Richardson and Bult 2015). The human Gene Ontology association (GOA) files for October 2020 and March 2021 VLAD analysis were downloaded from https://www.ebi.ac.uk/GOA/downloads.

## 3 Results

### 3.1 Gene Ontology Annotation

In October 2020, there was a lack of CCS development-relevant GO annotations describing 35 key CCS development human proteins (van Weerd and Christoffels 2016). 180 heart development GO terms were associated with these proteins, but of these only three manual annotations captured their role in CCS development (Table 1). Consequently, the priority for this annotation project was to use the GO to capture the contribution of these 35 proteins to CCS development. Due to time constraints, only 14 priority proteins were selected, from this list, for this focused annotation project. The majority of the experimental data available for these proteins used conditional gene knockout or overexpression mouse models. Therefore, many of the experimentally supported annotations created were associated with mouse or zebrafish proteins, these annotations were then copied to the equivalent human orthologs, supported by the ISS evidence code (Balakrishnan et al., 2013). Literature describing the role of the 14 priority proteins (Supplementary Tables S1, S2) was identified and curated, using appropriate GO terms (refer to Supplementary Table S3 for the full details of the annotations). A total of 99 GO annotations were created as a result of annotating 15 papers (https://tinyurl.com/puxypjyv, Supplementary Table S3), with 53 annotations copied to the human orthologs. These 15 articles supported the creation of 47 heart development annotations, 15 of which used CCS development terms. The remaining annotations captured the role of these proteins in regulating a variety of processes including cardiac conduction, gap junction assembly, heart rate, membrane depolarization, membrane repolarization and gene expression. A full list of 400 heart development GO annotations associated with the 35 key CCS development proteins can be found on https://tinyurl.com/2h8wutmy. In October 2020, in the human proteome only seven CCS development terms had been manually associated with five proteins. This project has increased this number to 49 annotations, associated with 33 proteins (Table 1). In addition, only 18 of the 35 key CCS development proteins were previously associated with a heart development GO term, now GJD3 is the only one of these proteins not associated with this GO biological process (March 2021). Human GJD3 is not associated with this process because mouse and human Gjd3 and GJD3 orthologs are not functionally equivalent, only the mouse protein plays a role in the AVN, the human protein does not (Kreuzberg et al., 2009). Consequently, we have prevented the CCS-relevant GO terms associated with mouse Gjd3 from being copied to human GJD3 by creating the equivalent annotations with the NOT qualifier (Feuermann et al., 2016).

**TABLE 1:** Impact of cardiac conduction-focused annotation project.

GO term identifier	GO term	Number of annotations March 2021		Number of annotations October 2020	
		All	Priority	All	Priority
GO:0003161	Cardiac conduction system development	17 (Gros et al., 2010)	17 (Gros et al., 2010)	3 (van Weerd and Christoffels, 2016)	3 (van Weerd and Christoffels, 2016)
GO:0003162	Atrioventricular node development	9 (Mommersteeg et al., 2007)	7 (Hoogaars et al., 2007)	2 (Kennedy et al., 2016)	0
GO:0003163	Sinoatrial node development	13 (Tessadori et al., 2012)	12 (Puskaric et al., 2010)	1 (Kennedy et al., 2016)	0
GO:0003164	His-Purkinje system development	10 (Hoogaars et al., 2007)	9 (Wiese et al., 2009)	1 (Kennedy et al., 2016)	0
Total number of CCS development annotations		49 (Richardson and Bult, 2015)	45 (Balakrishnan et al., 2013)	7 (Wiese et al., 2009)	3 (van Weerd and Christoffels, 2016)
GO:0007507	Heart development	1067 (356)	200 (Pashmforoush et al., 2004)	1023 (343)	177 (Hoogaars, 2004)
GO:0036302	Atrioventricular canal development	15 (Gaussin et al., 2002)	10 (Mommersteeg et al., 2007)	8 (Puskaric et al., 2010)	3 (3)
Total number of heart development GO annotations		1131 (373)	255 (Kreuzberg et al., 2009)	1038 (345)	183 (Hoogaars, 2004)

Comparison of the number of CCS and heart development manual GO annotations associated with all human gene products (All) and 35 key CCS development proteins in March 2021 (end of project) versus October 2020 (start of project). The six listed GO terms were used as a GO slim to filter the annotations downloaded from QuickGO. Full details of the annotations can be found in Supplementary Table S2 and https://tinyurl.com/3259w2vc and https://tinyurl.com/3525rt4u. The number of manual annotations associated with the human gene products are listed, with the number of gene products associated with these terms in brackets. The IEA (Inferred from Electronic Annotation) annotations have not been included as this data is updated with each release.

The review by van Weerd and Christoffels (2016) (van Weerd and Christoffels 2016) described 35 key CCS development proteins. Although we have associated CCS development terms with 30 of these proteins (Supplementary Table S2), for some of these proteins, the assertion that these proteins contribute to CCS development was based on expression data, which is not sufficient to meet the GO annotation guidelines. For these proteins, to meet the needs of the cardiovascular community and to represent expert knowledge, non-traceable author statement (NAS) annotations were created for the following 20 proteins: BMP4, CACN1AG, GATA4, GATA6, GJA1, GJB6, HCN4, HEY1, HEY2, HOPX, MSC, MSX1, MSX2, NOTCH2, NPPA, NPPB, SCN5A, SMAD1, SMAD4, and SMAD5 (indicated with asterisks (*) in Supplementary Table S2).

### 3.2 Delineation of Sinoatrial Node Development

Van Weerd and Christoffels (2016) (van Weerd and Christoffels, 2016) identified 10 proteins that are required for normal SAN development (BMP4, CACNA1G, HCN4, ISL1, NKX2-5, PITX2, SHOX2, TBX3, TBX5, and TBX18). Although van Weerd and Christoffels cited 19 articles as confirming the role of these proteins, only six of these articles provided the type of experimental support that met GO Consortium requirements and had not been previously curated with a CCS development GO term (Espinoza-Lewis et al., 2009; Espinoza-Lewis et al., 2011; Singh et al., 2012; Tessadori et al., 2012; Kapoor et al., 2013; Liang et al., 2015). The curation of these six articles led to the annotation of four proteins (ISL1, SHOX2, TBX3 and TBX18), therefore the Van Weerd and Christoffels review was used to support the association of the SAN development term with a further four proteins (BMP4, CACNA1G, HCN4 and GJB6). Two proteins (NKX2-5 and PITX2) inhibit SAN development and TBX5 has an ‘upstream’ role as it regulates SHOX2 expression in the inflow tract. Consequently, these three proteins were not associated with SAN development GO terms.

It is essential that the transcription factor Nkx2-5 is downregulated in cells which will develop into the SAN as shown by Espinoza-Lewis et al. (2009, 2011) (Espinoza-Lewis et al., 2009; Espinoza-Lewis et al., 2011) who reported ectopic expression of Nkx2-5 in the mouse heart resulted in hypoplastic SAN (resembling the Shox2−/− phenotype) (Espinoza-Lewis et al., 2009; Espinoza-Lewis et al., 2011). Former experimental evidence demonstrated that expression of Shox2 downregulates Nkx2-5 and led to the notion that Shox2 is an essential transcription factor required for correct development of the SAN and that Shox2 prevents the SAN from becoming the working myocardium by acting upstream of Nkx2-5 (Espinoza-Lewis et al., 2009; Espinoza-Lewis et al., 2011). This data was captured with GO terms describing the role of Shox2 in SAN development by associating the following annotations with Shox2: involved_in negative regulation of transcription by RNA polymerase II (GO:0000122) and involved_in sinoatrial node cell development (GO:0060931). Furthermore, these articles provided evidence for the role of Nkx2-5 in cardiac muscle tissue morphogenesis (GO:0055008) and cardiac muscle cell development (GO:0055013, Table 2). These authors also showed that Shox2 knockout mice were embryonic lethal due to bradycardia, which was associated with hypoplastic SAN and sinus valves (Espinoza-Lewis et al., 2009). Thus, additional annotations describing the role of Shox2 in heart development were provided based on Espinoza-Lewis et al. (2009) (Espinoza-Lewis et al., 2009) (Table 2). The role of ISL1 in SAN development was supported by zebrafish knockout data. Tessadori et al. (2012) showed that zebrafish embryos lacking isl1a protein exhibit bradycardia (Tessadori et al., 2012). This data supported the association of the zebrafish isl1a with the GO term sinoatrial node cell development (GO:0060931). Similarly, this GO term was also applied to the murine Isl1 protein during the curation of Liang et al. (2015) (Liang et al., 2015). The Isl1 mutant mouse data presented by Liang et al. also supported the creation of the GO annotation: Isl1 acts_upstream_of regulation of heart rate by cardiac conduction (GO:0086091). The GO term relation acts upstream of was selected because Isl1 does not directly regulate the heart rate. Kapoor et al. (2013) confirmed that adenoviral transfection of human TBX18 converts rodent ventricular myocytes to bona fide replicas of SAN pacemaker cells *in vitro* and *in vivo* (Kapoor et al., 2013). In addition, these authors also provided evidence that the TBX18 protein regulates the expression of the one of the hyperpolarization-activated cyclic nucleotide-gated channels (HCN4). This data led to three TBX18 annotations including TBX18 is involved_in sinoatrial node cell fate commitment (GO:0060930) and acts_upstream_of regulation of sinoatrial node cell action potential (GO:0098907).

**TABLE 2:** A selection of GO annotations describing the role of murine Shox2 and Nkx2-5 in heart development.

Protein	Qualifier	GO term	GO term identifier	Evidence code	Annotation extension	
					Relation	Identifier
Nkx2-5	Involved in	Cardiac muscle cell development	GO:0055013	IMP	—	—
NKx2-5	Involved in	Cardiac muscle tissue morphogenesis	GO:0055008	IMP	—	—
Shox2	Acts upstream of	Regulation of heart rate	GO:0002027	IMP	—	—
Shox2	Involved in	Sinoatrial node development	GO:0003163	IMP	—	—
Shox2	Involved in	Sinoatrial node cell development	GO:0060931	IMP	—	—
Shox2	Involved in	Cardiac right atrium morphogenesis	GO:0003213	IMP	—	—
Shox2	Involved in	Cardiac pacemaker cell differentiation	GO:0060920	IMP	Occurs_in	UBERON:0002351
					Part_of	GO:0003163
Shox2	Involved in	Negative regulation of transcription by RNA polymerase II	GO:0000122	IMP	Has_input	UniProtKB:P42582
					Occurs_in	UBERON:0002351

A selection of the GO terms and annotation extension statements used to capture the role of Shox2 and Nkx2-5, as described by Espinoza-Lewis et al. (2009 and 2011) (Espinoza-Lewis et al., 2009; Espinoza-Lewis et al., 2011). These GO terms had not been previously associated with these proteins. Terms associated with the listed annotation extension identifiers: Uberon:0002351, sinoatrial node; GO:0003163, sinoatrial node development; UniProtKB:P42582, Nkx2-5.

### 3.3 Delineation of AVC and Atrioventricular Node Development

Several members of the TBX transcription factor family are key players in both heart and CCS development including TBX2 and TBX3. TBX2 and TBX3 are important for the formation and patterning of the AVC, through repression of the working myocardial gene program (van Weerd and Christoffels 2016; Singh et al., 2012; Mohan et al., 2018). The AVN is derived from Tbx2+ cells (Aanhaanen et al., 2009) that originate in the AVC. Consequently, proteins involved in normal development of the AVC are directly or indirectly involved in the development of the AVN. Singh et al. (2012) showed that both TBX2 and TBX3 proteins are associated with early heart development processes, as overexpression of these human proteins in transgenic mice induced atrioventricular myocardial and endocardial cushion formation (Singh et al., 2012). This data was captured with 20 GO annotations, 16 of which were for the human TBX2 and TBX3 proteins, including TBX2 and TBX3 being involved in AVC development and AVC morphogenesis (Table 3). In addition, Moskowitz et al. (2004) demonstrated that Tbx5 knockout mice display CCS abnormalities including atrioventricular conduction delay (Moskowitz et al., 2004). Although this article had been curated previously, providing two Tbx5 annotations (pattern specification process (GO:0007389) and heart development (GO:0006507)), the involvement of the Tbx5 protein in CCS development had not been captured. This focused annotation project led to the addition of four Tbx5 annotations using the GO terms: atrioventricular bundle cell differentiation (GO:0003167), atrioventricular node cell development (GO:0060928), atrioventricular node cell fate commitment (GO:0060929), and positive regulation of cell communication by electrical coupling involved in cardiac conduction (GO:1901846).

**TABLE 3:** A selection of GO annotations describing the role of human TBX2 and TBX3 proteins in heart development.

Protein	Qualifier	GO term	GO term Identifier	Evidence code	With	Annotation extension	
						Relation	Identifier
Biological process							
TBX2	Involved in	Endocardial cushion formation	GO:0003272	IMP	—	—	—
TBX2	Involved in	Atrioventricular canal development	GO:0036302	ISS	Q60707 murine Tbx2	—	—
TBX2	Involved in	Atrioventricular canal morphogenesis	GO:1905222	ISS	Q60707 murine Tbx2	—	—
TBX2	Involved in	Cardiac jelly development	GO:1905072	IMP	—	—	—
TBX3	Involved in	Sinoatrial node cell development	GO:0060931	IDA	—	—	—
TBX3	Involved in	Endocardial cushion formation	GO:0003272	IMP	—	—	—
TBX3	Involved in	Atrioventricular canal development	GO:0036302	ISS	P70324 murine Tbx3	—	—
TBX3	Involved in	Atrioventricular canal morphogenesis	GO:1905222	ISS	P70324 murine Tbx3	—	—
TBX3	Involved in	Cardiac jelly development	GO:1905072	IMP	—	—	—
TBX3	Involved in	Cardiac epithelial to mesenchymal transition	GO:0060317	IMP	—	—	—
TBX3	Involved in	Negative regulation of cell proliferation involved in heart morphogenesis	GO:2000137	IMP	—	—	—
TBX3	Involved in	negative regulation of transcription by RNA polymerase II	GO:0000122	IMP	—	Occurs in	UBERON:0000948
						Part of	GO:0060317
TBX3	Involved in	positive regulation of transcription by RNA polymerase II	GO:0000315	IMP	—	Occurs in	UBERON:0000948
						Part of	GO:0060317
Molecular function							
TBX3	Enables	RNA polymerase II *cis*-regulatory region sequence-specific DNA binding	GO:0000978	IDA	—	Occurs in	UBERON:0000948
TBX3	Enables	DNA-binding transcription activator activity, RNA polymerase II-specific	GO:0001228	IDA	—	Occurs in	UBERON:0000948
						Part of	GO:0060317
TBX3	Enables	DNA-binding transcription repressor activity, RNA polymerase II-specific	GO:0001227	IDA	---	Occurs in	UBERON:0000948
						Part of	GO:0060317

Twenty GO annotations were created following the review of Singh et al. (2012) (Singh et al., 2012). Sixteen of these annotations describe the role of human TBX2 and TBX3 in heart development. Terms associated with the listed annotation extension identifiers: Uberon: 0000948, heart; GO:0060317, cardiac epithelial to mesenchymal transition.

Cai et al. (2011) (Cai et al., 2011) found that Tbx20 conditional knockout mice did not survive to E10.5 and displayed mutant hearts with reduced AVC constriction when compared to the heterozygous, normal littermates (Cai et al., 2011). The mutants exhibited scarce mesenchymal cells and disrupted AVC cushion mesenchyme formation, which led to the following annotations to capture the role of Tbx20: involved_in atrioventricular valve development (GO:0003171), endocardial cushion formation (GO:0003272), positive regulation of epithelial to mesenchymal transition (GO:0010718), mesenchymal cell development (GO:0014031), positive regulation of BMP signaling pathway (GO:0030513), and atrioventricular canal development (GO:0036302).

Bone morphogenic proteins (BMPs) are important for regulating atrioventricular canal (AVC) development in vertebrates. In mice, myocardial Tbx20 is required for early AVC development and upregulation of Bmp2 (Cai et al., 2011) and Bmp10 (van Weerd and Christoffels 2016). As Bmp2 expression was significantly downregulated in Tbx20 conditional knockout mouse hearts, Cai et al. (2011) examined the effect of overexpressing Bmp2 in these mice and found that the mesenchymal defects associated with a lack of Tbx20 protein were substantially rescued (Cai et al., 2011). Thus, the following annotation was made for Bmp2: involved_in epithelial to mesenchymal transition (GO:0001837).

### 3.4 Delineation of Ventricular Conduction System Development

Following this focused annotation project, six proteins are now associated with GO terms describing His-Purkinje system development or development of part of the VCS: HOPX, ID2, IRX3, NKX2-5, TBX3, and TBX5. Four of these were previously associated with this term, with IEA evidence, and this project has added two proteins, IRX3 and HOPX, to this GO group.

IRX3 is a transcription factor expressed in the trabecular ventricular myocardium and is known to be relevant to direct suppression of GJA1 and indirect activation of GJA5 and SCN5A (Christoffels et al., 2000; Zhang et al., 2011; Koizumi et al., 2016). GO terms were associated with the murine Irx3 protein (Supplementary Table S4) based on the experimental evidence described by Zhang et al. (2011) which found that the loss of Irx3 is associated with increased conduction time between the bundle of His and ventricles (Zhang et al., 2011), without affecting conduction from the atria to the bundle of His. The key effects of this transcription factor are regulation of gap junction protein expression, increasing Gja5 and decreasing Gja1. The loss of Irx3 impacted the assembly of gap junctions which led to changes in the electrical coupling of the CCS and ultimately disruption of the coordinated spread of ventricular excitation. Based on this, the annotations associated with Irx3 describe its role in the following biological processes: positive regulation of gap junction assembly (GO:1903598), negative regulation of transcription by RNA polymerase II (GO:0000122) occurs in UBERON has_input, positive regulation of transcription of RNA polymerase II (GO:0045944), regulation of cell communication by electrical coupling involved in cardiac conduction (GO:1901844). The functional annotations associated with Irx3 include RNA polymerase II cis-regulatory region sequence-specific DNA binding (GO:0000978), DNA-binding transcription activator activity (GO:0001216), DNA-binding transcription repressor activity, RNA polymerase II-specific (GO:0001227)*. Irx3* ^*+/−*^mice were crossed with Gja5+/EGFP knock-in reporter mice that express GFP under the control of the Gja5 promoter (Kim et al., 2016). Morphological defects were observed in the *Irx3−/−* mice such as significant reductions in the VCS fiber densities throughout the ventricles and absence of the RBB. Aberrant electrical activation of the ventricles was observed in these mice and exhibited prolonged QRS intervals with R notches in ECG and abnormal activation patterns with RBB block in optical mapping. These findings were captured using the following GO annotations: Irx3 is involved_in Purkinje myocyte development (GO:0003165), atrioventricular bundle cell differentiation (GO:0003167), His-Purkinje system cell differentiation (GO:0060932), and positive regulation of gap junction assembly (GO:1903598) occurring in the His-Purkinje system.

### 3.5 Functional Enrichment Analysis

All manual gene annotations made during this study are publicly available (Supplementary Table S3: https://tinyurl.com/puxypjyv) and were included in the functional enrichment tool VLAD to test for the enrichment of ontology terms in the Query Set of 35 key CCS development proteins (Supplementary Table S2). The results of the VLAD functional analysis are depicted in Supplementary Figure S1, where the node sizes are scaled based on the *p*-values. Table 4 shows the VLAD statistical analysis of six GO terms used with the graphical output presented in Supplementary Figure S1. In October 2020, 13 human proteins were associated with the CCS development GO terms (8 of which were supported with IEA evidence, Table 4). In March 2021, this number increased to 38 and 30, respectively (this total includes IEA annotations, Table 4). The SAN development term has an additional 7 proteins associated with it and the AVN development term has an additional 4 proteins associated. Overall, in both functional enrichment analyses there was a significant enrichment of these GO terms, but the *p*-value was considerably more significant with the inclusion of the GO annotation data present in March 2021 compared to that of October 2020.

**TABLE 4:** Impact of this focused annotation project on functional enrichment analysis.

	GO term identifier	GO term name	P	k	K	K/n (%)	K/K (%)	Proteins associated with enriched term (HGNC symbols)
October 2020 (*N* = 17572)	GO:0003161	cardiac conduction system development	6.75E-14	6	13	17.14	46.15	BMPR1A, GJA5, ID2, NKX2-5, TBX3, TBX5
	GO:0003164	His-Purkinje system development	6.58E-11	4	5	11.43	80.00	ID2, NKX2-5, TBX3, TBX5
	GO:0003162	atrioventricular node development	5.96E-03	1	3	2.86	33.33	NKX2-5
	GO:0003163	sinoatrial node development	5.96E-03	1	3	2.86	33.33	TBX3
	GO:0036302	atrioventricular canal development	6.03E-07	3	9	8.57	33.33	BMP2, SMAD4, TBX2
	GO:0007507	heart development	3.55E-31	25	514	71.43	4.86	BMP2, BMP4, BMPR1A, GATA4, GATA6, GJA1, GJA5, HEY1, HEY2, ID2, ISL1, MSX1, MSX2, NKX2-5, NOTCH2, NPPA, PITX2, SCN5A, SHOX2, SMAD1, SMAD4, TBX2, TBX3, TBX5, TBX20
March 2021 (*N* = 17655)	GO:0003161	cardiac conduction system development	1.69E-82	30	38	85.71	78.95	BMP4, BMPR1A, CACNA1G, GATA4, GATA6, GJA1, GJA5, GJB6, HCN4, HEY1, HEY2, HOPX, ID2, IRX3, ISL1, MSC, MSX1, MSX2, NKX2-5, NOTCH2, NPPA, NPPB, SCN5A, SHOX2, SMAD1, SMAD4, SMAD5, TBX3, TBX5, TBX18
	GO:0003164	His-Purkinje system development	2.27E-14	5	5	14.29	100.00	HOPX, ID2, IRX3, NKX2-5, TBX3, TBX5
	GO:0003162	atrioventricular node development	4.76E-13	5	7	14.29	71.43	BMPR1A, GATA4, GATA6, NKX2-5 NOTCH2, TBX5
	GO:0003163	sinoatrial node development	4.52E-21	8	10	22.86	80.00	BMP4, CACNA1G, GJB6, HCN4, ISL1, SHOX2, TBX3 TBX18
	GO:0036302	atrioventricular canal development	1.08E-16	7	13	20.00	53.85	BMP2, GATA4, GATA6, SMAD4, TBX2, TBX3, TBX20
	GO:0007507	heart development	7.96E-52	34	516	97.14	6.59	BMP2, BMP4, BMPR1A, CACNA1G, GATA4, GATA6, GJA1, GJA5, GJB6, HCN4, HEY1, HEY2, HOPX, ID2, IRX3, ISL1, MSC, MSX1, MSX2, NKX2-5, NOTCH2, NPPA, NPPB, PITX2, SCN5A, SHOX2, SMAD1, SMAD4, SMAD5, TBX18, TBX2, TBX3, TBX5, TBX20

VisuaL Annotation Display (VLAD) statistical analysis for the two datasets (October 2020 and March 2021) based on the six GO slim terms used in the graphical output shown in Figure 1. The total number of annotated human proteins (N) is 17572 and 17655 for October 2020 and March 2021, respectively, with 35 key CCS development proteins in the Query Set (n). K is the number of human proteins associated with the listed GO term or its descendants and k is the number of proteins in the Query Set associated with the GO term or its descendants.

## 4 Discussion

SAN and other CCS dysfunctions can occur upon ageing and disease, including sick sinus syndrome, which can lead to higher rates of pacemaker implantation (Dobrzynski et al., 2007). This study aimed to improve our understanding of the complex genetic pathways involved in CCS development and describe the specific roles of 35 key CCS development proteins (Supplementary Tables S1, S2) that were reported in the review by van Weerd and Christoffels (2016) (van Weerd and Christoffels 2016). The review of 15 articles during this focused annotation approach led to the creation of 99 annotations, 47 of which were then copied to the orthologous human protein record. The majority of these annotations captured the role of the 14 priority proteins in the development of the CCS or other heart tissue, but some of these annotations captured the role of these proteins in other CCS-relevant processes, such as the role of murine Gjd3 in electrical coupling and the regulation of transcription by IRX3, SHOX2, TBX3, TBX5. In addition, GO terms describing epithelial to mesenchymal transition, regulation of heart rate and cell-cell signaling involved in cardiac conduction, as well as molecular function terms, such as DNA-binding transcription activator activity, RNA polymerase II-specific and gap junction channel activity involved in bundle of His cell-Purkinje myocyte electrical coupling, were also used.

Although the main aim of this study was to manually annotate the proteins involved in CCS development, curation of other development terms arose. The transcription factor PITX2 does not play a direct role in SAN development, but does have an essential role in the early events of left-right determination (Franco et al., 2014). Pitx2 knockout mice confirm the importance of Pitx2 for normal development of the heart, these mice have two SANs and display features of right isomerism. This phenotype supported the following annotation for Pitx2: involved_in embryonic heart tube left/right pattern formation (GO:0060971). As the primary role of Pitx2 is in determining the asymmetry of the heart, in line with GO Consortium guidelines, the downstream impact of Pitx2 knockout on SAN development was not captured.

Although 35 proteins were described as being involved in the formation and function of the CCS, applying the GO consortium guidelines meant that only 15 peer-reviewed primary experimental articles could be curated by using carefully selected descriptive GO terms. Therefore, the role of 20 proteins in CCS development was captured based on author statements, and by doing so, we were able to curate information that would not otherwise be available. It is important to remember that there is a discrepancy of data interpretation between developmental biologists and Gene Ontology biocurators regarding the robustness of expression data. For example, experimental biologists may interpret data describing the location of expression of a transcription factor as an indicator of its function at that location, whereas this data articles is not usually considered to be experimentally robust enough to satisfy the GO Consortium requirements for curation.

Despite a focused approach to curate the key proteins involved in CCS development, it is evident that the GO annotation datasets are still incomplete, however, without a full review of the literature, it is difficult to predict how many more proteins should be associated with CCS development GO terms. There are many different knowledgebases that provide information about the genes and proteins that are required for correct development of the CCS. Open Targets (Ochoa et al., 2021) provides a list of nearly 2000 genes associated with “genetic cardiac rhythm disease,” of which 151 are identified by genetic interaction data provided by a variety of manually created resources, including UniProt (The UniProt Consortium et al., 2021), ClinVar (Landrum et al., 2020) and PanelApp (Martin et al., 2019). While the Mouse Genome Informatics (MGI) (Ringwald et al., 2021) identifies 12 genes associated with “abnormal impulse conducting system morphology” and 53 genes associated with “heart conduction disorder.” A search using the Online Mendelian Inheritance in Man (OMIM) database (McKusick 2021), for the term “conduction” resulted in a list of 183 entries (16 April 2021), implying there are many CCS-related diseases. Surprisingly, only 8 of the proteins listed in van Weerden and Christoffels review are also included in the OMIM list of conduction entries: GATA4, GJA1, GJA5, ID2, NKX2-5, NOTCH2, SCN5A, and TBX20. This confirms that the GO annotation files have captured only the tip of the iceberg of the proteins that need to be associated with CCS development terms. For example, variants in protein kinase AMP-activated non-catalytic subunit gamma 2 (PRKAG2) has been identified as causative of familial Wolff-Parkinson-White Syndrome (Miyamoto 2018), but no CCS-related annotations are associated with this gene, even though these patients exhibit short PR interval, prolonged QRS and have ectopic conductive atrioventricular (accessory) pathways. Considerably more work is, therefore, required to curate existing experimental and phenotype data that describes the role of all cardiac conduction disease-associated genes.

Although, a functional enrichment analysis of the 35 key CCS development proteins confirmed that this work has improved the *in-silico* interpretation of this CCS dataset, researchers should be cautious when interpreting GO enrichment analyses due to annotation bias and incomplete annotation of the accumulated biomedical corpus (Tomczak et al., 2018). However, focused annotation approaches, such as this, do provide an opportunity for the creation of more detailed annotations that are often missed when a broad annotation approach is taken.

Overall, this focused annotation project has made an important contribution to the GO annotation dataset by increasing the total number of CCS development annotations. The project has expanded the available GO description of CCS development and heart development by creating 72 new heart development annotations, 42 of which described CCS development. This was achieved by the manual curation of 14 priority proteins and the addition of author statement (NAS) supported annotations associated with a further 20 proteins. Our contribution to the GO annotation dataset may help elucidate the key mechanisms involved in various CCS disorders, as previous annotation projects have focused predominantly on development of the heart rather than the CCS. Further work is needed to ensure comprehensive annotation of every gene associated with cardiac conduction abnormalities.

## Data Availability

The datasets presented in this study can be found in online repositories. The names of the repository/repositories and accession number(s) can be found in the article/Supplementary Material.
