# Supplementary material for: GOing Forward With the Cardiac Conduction System Using Gene Ontology
Source: Front Genet. 2022 Mar 2;13:802393. doi: 10.3389/fgene.2022.802393 (PMC8924464; doi:10.3389/fgene.2022.802393)
Supplement: Supplementary file 1 [file DataSheet1.docx]

Supplementary Material

**Supplementary Table S1. Key cardiac conduction system development human proteins.** The list of 35 proteins described as having a role in the development of the cardiac conduction system (2). The priority proteins are indicated.

| **HGNC gene symbol, name (with link to UniProt record)** | **Brief description of protein function** | **Priority proteins** | **UniProt ID** |
| --- | --- | --- | --- |
| [*BMP2*](https://www.uniprot.org/uniprot/P12643), bone morphogenic protein 2 | Growth factor of the TGF-beta superfamily | Priority | P12643 |
| [*BMP4*](https://www.uniprot.org/uniprot/P12644), bone morphogenic protein 4 | Growth factor of the TGF-beta superfamily |  | P12644 |
| [*BMPR1A*](https://www.uniprot.org/uniprot/P36894), bone morphogenic protein receptor type 1A | Growth factor signaling receptor | Priority | P36894 |
| [*CACNA1G*](https://www.uniprot.org/uniprot/O43497), calcium voltage-gated channel subunit alpha1 G | Calcium voltage-gated channel subunit |  | O43497 |
| [*GATA4*](https://www.uniprot.org/uniprot/P43694), GATA binding protein 4 | DNA binding transcription factor |  | P43694 |
| [*GATA6*](https://www.uniprot.org/uniprot/Q92908), GATA binding protein 6 | DNA binding transcription factor |  | Q92908 |
| [*GJA1*](https://www.uniprot.org/uniprot/P17302), gap junction protein alpha 1 | Gap junction transmembrane channel subunit |  | P17302 |
| [*GJA5*](https://www.uniprot.org/uniprot/P36382), gap junction protein alpha 5 | Gap junction transmembrane channel subunit | Priority | P36382 |
| [*GJB6*](https://www.uniprot.org/uniprot/O95452), gap junction protein beta 6 | Gap junction transmembrane channel subunit |  | O95452 |
| [*GJD3*](https://www.uniprot.org/uniprot/Q8N144), gap junction protein delta 3 | Gap junction transmembrane channel subunit | Priority | Q8N144 |
| [*HCN4*](https://www.uniprot.org/uniprot/Q9Y3Q4), hyperpolarization activated cyclic nucleotide gated potassium channel 4 | Calcium voltage-gated channel subunit |  | Q9Y3Q4 |
| [*HEY1*](https://www.uniprot.org/uniprot/Q9Y5J3), hes related family bHLH transcription factor with YRPW motif 1 | DNA binding transcription factor |  | Q9Y5J3 |
| [*HEY2*](https://www.uniprot.org/uniprot/Q9UBP5), hes related family bHLH transcription factor with YRPW motif 2 | DNA binding transcription factor |  | Q9UBP5 |
| [*HOPX*](https://www.uniprot.org/uniprot/Q9BPY8), HOP homeobox | DNA binding transcription factor |  | Q9BPY8 |
| [*ID2*](https://www.uniprot.org/uniprot/Q02363), inhibitor of DNA binding 2 | DNA binding transcription factor inhibitor |  | Q02363 |
| [*IRX3*](https://www.uniprot.org/uniprot/P78415), iroquois homeobox 3 | DNA binding transcription factor | Priority | P78415 |
| [*ISL1*](https://www.uniprot.org/uniprot/P61371), ISL LIM homeobox 1 | DNA binding transcription factor | Priority | P61371 |
| [*MSC*](https://www.uniprot.org/uniprot/O60682), musculin | DNA binding transcription factor |  | O60682 |
| [*MSX1*](https://www.uniprot.org/uniprot/P28360), msh homeobox 1 | DNA binding transcription factor |  | P28360 |
| [*MSX2*](https://www.uniprot.org/uniprot/P35548), msh homeobox 1 | DNA binding transcription factor |  | P35548 |
| [*NKX2-5*](https://www.uniprot.org/uniprot/P52952)*,* NK2 homeobox 5 | DNA binding transcription factor | Priority | P52952 |
| [*NOTCH2*](https://www.uniprot.org/uniprot/Q04721), notch receptor 2 | Signaling receptor and transcription co-activator |  | Q04721 |
| [*NPPA*](https://www.uniprot.org/uniprot/P01160), natriuretic peptide A | Hormone |  | P01160 |
| [*NPPB*](https://www.uniprot.org/uniprot/P16860), natriuretic peptide B | Hormone |  | P16860 |
| [*PITX2*](https://www.uniprot.org/uniprot/Q99697), paired like homeodomain 2 | DNA binding transcription factor | Priority | Q99697 |
| [*SCN5A*](https://www.uniprot.org/uniprot/Q14524), sodium voltage-gated channel alpha subunit 5 | Sodium voltage-gated channel subunit |  | Q14524 |
| [*SHOX2*](https://www.uniprot.org/uniprot/O60902), short stature homeobox 2 | DNA binding transcription factor | Priority | O60902 |
| [*SMAD1*](https://www.uniprot.org/uniprot/Q15797), SMAD family member 1 | Signaling molecule |  | Q15797 |
| [*SMAD4*](https://www.uniprot.org/uniprot/Q13485)*,* SMAD family member 4 | Signaling molecule and transcription co-activator |  | Q13485 |
| [*SMAD5*](https://www.uniprot.org/uniprot/Q99717), SMAD family member 5 | Signaling molecule and transcription co-activator |  | Q99717 |
| [*TBX2*](https://www.uniprot.org/uniprot/Q13207), T-box transcription factor 2 | DNA binding transcription factor | Priority | Q13207 |
| [*TBX3*](https://www.uniprot.org/uniprot/O15119), T-box transcription factor 3 | DNA binding transcription factor | Priority | O15119 |
| [*TBX5*](https://www.uniprot.org/uniprot/Q99593), T-box transcription factor 5 | DNA binding transcription factor | Priority | Q99593 |
| [*TBX18*](https://www.uniprot.org/uniprot/O95935), T-box transcription factor 18 | DNA binding transcription factor | Priority | O95935 |
| [*TBX20*](https://www.uniprot.org/uniprot/Q9UMR3), T-box transcription factor 20 | DNA binding transcription factor | Priority | Q9UMR3 |

**Supplementary Table S2. The number of annotations associated with the key cardiac conduction system development human proteins.** All GO annotations associated with 35 key cardiac conduction system (CCS) development proteins identified by van Weerden and Christoffels (2). 14 of these proteins were prioritized for curation in this study (highlighted in purple). The number of CCS development-relevant annotations currently associated with these proteins were identified using six GO terms as GO slims (Figure 1): CCS development, atrioventricular node (AVN) development, sinoatrial node (SAN) development, atrioventricular canal (AVC) development, His-Purkinje system development, and heart development. The GO slim filter extracts annotations either directly to the term or a child of the term (unless the child term is also included in the GO slim). Numbers in brackets indicate the number of annotations created by direct review of the literature (i.e. excluding IEA (Inferred from Electric Annotation) and IBA (Inferred from Biological aspect of Ancestor) evidence). 20 non-traceable author statement (NAS) annotations based on the van Weerden and Christoffels (2016) review (2) were created for 20 proteins (indicated with asterisks (*)): BMP4, CACNA1G, GATA4, GATA6, GJA1, GJB6, HCN4, HEY1, HEY2, HOPX, MSC, MSX1, MSX2, NPPA, NPPB, NOTCH2, SCN5A, SMAD1, SMAD4, and SMAD5 (data downloaded from QuickGO (32) on 10 May 2021).

| **Protein** | **Number of annotations associated with cardiac conduction system GO slim terms** | | | | **Total number of CCS development annotations** | **Number of GO:0007507  heart development annotations** | **Number of GO:0036302  AVC development annotations** | **Total number of annotations to a heart development GO term** |
| --- | --- | --- | --- | --- | --- | --- | --- | --- |
|  | **GO:0003161  CCS development** | **GO:0003162  AVN development** | **GO:0003163  SAN development** | **GO:0003164 His-Purkinje system development** |  |  |  |  |
| BMP2 | 0 | 0 | 0 | 0 | 0 | 27 (16) | 2 (1) | 29 (17) |
| BMP4 | 0 | 0 | 1 (1) | 0 | 1* | 28 (16) | 0 | 29 (17) |
| BMPR1A | 2 (1) | 2 (1) | 0 | 0 | 4 | 36 (18) | 0 | 40 (20) |
| CACNA1G | 0 | 0 | 1 (1) | 0 | 1* | 0 | 0 | 1 (1) |
| GATA4 | 0 | 1 (1) | 0 | 0 | 1* | 15 (13) | 1 (1) | 17 (15) |
| GATA6 | 0 | 1 (1) | 0 | 0 | 1* | 7 (3) | 1 (1) | 9 (5) |
| GJA1 | 1 (1) | 0 | 0 | 0 | 1* | 3 (0) | 0 | 4 (1) |
| GJA5 | 1 (1) | 0 | 0 | 0 | 1 | 6 (5) | 0 | 7 (6) |
| GJB6 | 0 | 0 | 1 (1) | 0 | 1* | 0 | 0 | 1 (1) |
| GJD3 | 0 | 0 | 0 | 0 | 0 | 0 | 0 | 0 |
| HCN4 | 0 | 0 | 1 (1) | 0 | 1* | 0 | 0 | 2 (1) |
| HEY1 | 1 (1) | 0 | 0 | 0 | 1* | 10 (10) | 0 | 11 (11) |
| HEY2 | 1 (1) | 0 | 0 | 0 | 1* | 40 (18) | 0 | 41 (19) |
| HOPX | 0 | 0 | 0 | 1 (1) | 1* | 1 (0) | 0 | 2 (1) |
| ID2 | 0 | 0 | 0 | 1 (1) | 1 | 1 (1) | 0 | 2 (2) |
| IRX3 | 0 | 0 | 0 | 6 (3) | 6 | 0 | 0 | 6 (3) |
| ISL1 | 0 | 0 | 3 (2) | 0 | 3 | 20 (9) | 0 | 23 (11) |
| MSC | 1 (1) | 0 | 0 | 0 | 1* | 0 | 0 | 1 (1) |
| MSX1 | 1 (1) | 0 | 0 | 0 | 1* | 4 (0) | 0 | 5 (1) |
| MSX2 | 1 (1) | 0 | 0 | 0 | 1* | 0 | 0 | 1 (1) |
| NKX2-5 | 1 (1) | 0 | 0 | 0 | 1 | 21 (20) | 0 | 22 (21) |
| NOTCH2 | 0 | 1 (1) | 0 | 0 | 1* | 4 (3) | 0 | 5 (4) |
| NPPA | 1 (1) | 0 | 0 | 0 | 1* | 2 (1) | 0 | 3 (2) |
| NPPB | 1 (1) | 0 | 0 | 0 | 1* | 0 | 0 | 1 (1) |
| PITX2 | 0 | 0 | 0 | 0 | 0 | 3 (3) | 0 | 3 (3) |
| SCN5A | 1 (1) | 0 | 0 | 0 | 1* | 1 (1) | 0 | 2 (2) |
| SHOX2 | 0 | 0 | 4 (2) | 0 | 4 | 6 (2) | 0 | 10 (4) |
| SMAD1 | 1 (1) | 0 | 0 | 0 | 1* | 1 (0) | 0 | 2 (1) |
| SMAD4 | 1 (1) | 0 | 0 | 0 | 1* | 16 (7) | 2 (1) | 19 (9) |
| SMAD5 | 1 (1) | 0 | 0 | 0 | 1* | 0 | 0 | 1 (1) |
| TBX2 | 0 | 0 | 0 | 0 | 0 | 20 (11) | 5 (3) | 25 (14) |
| TBX3 | 0 | 0 | 2 (1) | 1 (0) | 3 | 12 (3) | 4 (2) | 19 (6) |
| TBX5 | 0 | 4 (2) | 0 | 4 (2) | 8 | 22 (10) | 0 | 30 (14) |
| TBX18 | 0 | 0 | 3 (3) | 0 | 3 | 0 | 0 | 3 (3) |
| TBX20 | 0 | 0 | 0 | 0 | 0 | 41 (22) | 2 (1) | 43 (23) |
| **Grand Total** | **16 (15)** | **9 (6)** | **16 (12)** | **14 (8)** | **54** | **347 (192)** | **17 (10)** | **419 (242)** |

**Supplementary Table S3. Summary of the articles curated in this study based on a review by van Weerden and Christoffels (2016) (2).**A total of 152 descriptive GO annotations were submitted to the GO Consortium resource, of which 45 are associated with one of the heart development GO slim terms (Supplementary Table S2). The PubMed identifiers of the articles curated in this study are indicated and these annotations are available to view in GO browsers, such as QuickGO (32) (<https://tinyurl.com/puxypjyv>). The approved gene symbols are provided by HGNC (26), MGI (52) or ZFIN (https://zfin.org/) for human, mouse, and zebrafish proteins, respectively. Annotations associated with mouse or zebrafish proteins were copied to the human orthologous proteins, with the inferred by sequence similarity evidence code (ISS). The number of ISS annotation is indicated in brackets, note that if the equivalent GO term was already associated with a human protein, then the ISS annotation was not created.

| **PubMed Identifier** | **Protein** | **Species** | **GO term name** | **Number of GO annotations** |
| --- | --- | --- | --- | --- |
| 17234970 | Pitx2 | Mouse | Embryonic heart tube left/right pattern formation | 3 (3) |
|  | Nkx2.5 | Mouse | Atrial cardiac muscle tissue development  Heart morphogenesis |  |
| 21640717 | Nkx2.5 | Mouse | Cardiac muscle cell development  Cardiac muscle tissue morphogenesis  Negative regulation of transcription by RNA polymerase II  Sinoatrial node development | 4 (4) |
| 19166829 | Shox2 | Mouse | Cardiac pacemaker cell differentiation  Cardiac right atrium morphogenesis  Regulation of heart rate  Sinoatrial node development | 5 (4) |
|  | Nkx2.5 | Mouse | Cardiac muscle cell development |  |
| 23077655 | isl1a | Zebrafish | Sinoatrial node development | 1 (1) |
| 26193633 | Isl1 | Mouse | Regulation of heart rate by cardiac conduction  Sinoatrial node cell development | 2 (2) |
| 23242162 | TBX18 | Human | Regulation of SA node cell action potential  Sinoatrial node cell development  Sinoatrial node cell fate commitment | 3 (0) |
| 22130515 | Tbx2 | Mouse | Atrioventricular canal development  Atrioventricular canal morphogenesis | 20 (4) |
|  | TBX2 | Human | Atrioventricular canal development  Atrioventricular canal morphogenesis  Cardiac jelly development  Endocardial cushion formation |  |
|  | Tbx3 | Mouse | Atrioventricular canal development  Atrioventricular canal morphogenesis |  |
|  | TBX3 | Human | Atrioventricular canal development  Atrioventricular canal morphogenesis  Cardiac epithelial to mesenchymal transition  Cardiac jelly development  Endocardial cushion formation  Negative regulation of transcription by RNA polymerase II  Negative regulation of cell proliferation involved in heart morphogenesis  Positive regulation of transcription by RNA polymerase II  Sinoatrial node cell development  DNA-binding transcription activator activity  DNA-binding transcription repressor activity, RNA polymerase II-specific  RNA polymerase II cis-regulatory region sequence-specific DNA binding |  |
| 15289437 | Tbx5 | Mouse | Atrioventricular bundle cell differentiation  Atrioventricular node cell development  Atrioventricular node cell fate commitment | 4 (3) |
|  | TBX5 | Human | Positive regulation of cell communication by electrical coupling involved in cardiac conduction |  |
| 22728936 | Tbx5 | Mouse | Bundle of His cell to Purkinje myocyte communication by electrical coupling  Bundle of His development  Cell-cell signaling involved in cardiac conduction  Positive regulation of gap junction assembly  Positive regulation of cardiac conduction  Positive regulation of transcription by RNA polymerase II  Regulation of atrial cardiac muscle cell membrane depolarization  RNA polymerase II cis-regulatory region sequence-specific DNA binding  DNA-binding transcription activator activity, RNA polymerase II-specific | 9 (9) |
| 16571663 | Gjd3 | Mouse | AV node cell to bundle of His cell communication by electrical coupling  Negative regulation of cardiac conduction  Negative regulation of cell communication by electrical coupling involved in cardiac conduction  Negative regulation of heart rate | 4 (0) |
| 17998461 | Bmpr1a | Mouse | Atrioventricular node cell development | 1 (1) |
| 11854453 | Bmpr1a | Mouse | Atrioventricular valve development  BMP signaling pathway involved in heart development  Embryonic organ development  Endocardial cushion morphogenesis  Negative regulation of gene expression  Positive regulation of transforming growth factor beta2 production  Ventricular septum morphogenesis  Ventricular trabecula myocardium morphogenesis | 8 (8) |
| 21825130 | Irx3 | Mouse | Negative regulation of transcription by RNA polymerase II  Positive regulation of gap junction assembly  Positive regulation of transcription by RNA polymerase II Regulation of cell communication by electrical coupling involved in cardiac conduction  DNA-binding transcription activator activity  DNA-binding transcription repressor activity, RNA polymerase II-specific  RNA polymerase II cis-regulatory region sequence-specific DNA binding | 14 (7) |
|  | IRX3 | Human | Negative regulation of transcription by RNA polymerase II  Positive regulation of gap junction assembly  Positive regulation of transcription by RNA polymerase II  Regulation of cell communication by electrical coupling involved in cardiac conduction  DNA-binding transcription activator activity  DNA-binding transcription repressor activity, RNA polymerase II-specific  RNA polymerase II cis-regulatory region sequence-specific DNA binding |  |
| 26786475 | Gja5 | Mouse | Bundle of His cell to Purkinje myocyte communication by electrical coupling  Regulation of ventricular cardiac muscle cell membrane repolarization  Regulation of ventricular cardiac muscle cell membrane depolarization  Gap junction channel activity involved in bundle of His cell-Purkinje myocyte electrical coupling | 8 (8) |
|  | Irx3 | Mouse | Atrioventricular bundle cell differentiation  His-Purkinje system cell differentiation  Positive regulation of gap junction assembly  Purkinje myocyte development |  |
| 21983003 | Bmp2 | Mouse | Epithelial to mesenchymal transition | 7 (5) |
|  | Tbx20 | Mouse | Atrioventricular canal development  Atrioventricular valve development  Endocardial cushion formation  Mesenchymal cell development  Positive regulation of BMP signaling pathway  Positive regulation of epithelial to mesenchymal transition |  |

**Supplementary Table S4. A selection of GO terms and annotation extension statements used to curate the role of mouse Irx3 using experimental evidence described by Zhang et al. (2011) (44)**. The evidence code is the code used to support the annotation: IDA indicates Inferred from Direct Assay; IMP indicates Inferred from Mutant Phenotype.

| **Gene symbol** | **Qualifier** | **GO term name** | **GO ID** | **Evidence code** | **Annotation extension** | |
| --- | --- | --- | --- | --- | --- | --- |
|  |  |  |  |  | **Relation** | **Identifier and name** |
| **Biological process** | | | | | | |
| Irx3 | involved in | Negative regulation of transcription by RNA polymerase II | GO:0000122 | IDA | Occurs in | UBERON:0004146 His-Purkinje system |
|  |  |  |  |  | Has input | UniProtKB:P23242 Gja1 |
|  |  |  |  |  | Part of | GO:1903596 regulation of gap junction assembly |
| Irx3 | Acts upstream of | Regulation of cell communication by electrical coupling involved in cardiac conduction | GO:1901844 | IMP | Occurs in | UBERON:0004146 His-Purkinje system |
| Irx3 | Involved in | Positive regulation of gap junction assembly | GO:1903598 | IMP | Occurs in | UBERON:0004146 His-Purkinje system |
|  |  |  |  |  | Causally upstream of | GO:1901844 regulation of cell communication by electrical coupling involved in cardiac conduction |
| Irx3 | Involved in | Positive regulation of transcription by RNA polymerase II | GO:0045944 | IDA | Occurs in | UBERON:0002354 cardiac Purkinje fiber |
|  |  |  |  |  | Has input | UniProtKB:Q01231 Gja5 |
|  |  |  |  |  | Part of | GO:1903598 positive regulation of gap junction assembly |
| **Molecular function** | | | | | | |
| Irx3 | Enables | DNA-binding transcription activator activity, RNA polymerase II-specific | GO:0001228 | IDA | Occurs in | UBERON:0002354 cardiac Purkinje fiber |
|  |  |  |  |  | Has input | UniProtKB:Q01231 Gja5 |
|  |  |  |  |  | Part of | GO:1903598 positive regulation of gap junction assembly |
| Irx3 | Enables | DNA-binding transcription repressor activity, RNA polymerase II-specific | GO:0001227 | IDA | Occurs in | UBERON:0004146 His-Purkinje system |
|  |  |  |  |  | Has input | UniProtKB:P23242 Gja1 |
|  |  |  |  |  | Part of | GO:1903596 regulation of gap junction assembly |

**Supplementary Figure S1: Impact of this focused annotation project on functional enrichment analysis.** Graphical output of the VLAD biological process analysis comparing the March 2021 annotation dataset (top graph) with the October 2020 annotation dataset (lower graph). Each node represents a specific enriched GO term, with: GO ID; GO term name; p-value; k is the number of proteins in the query dataset associated with the term; K is the number of proteins in the human proteome associated with the term. The size of the node is scaled based on the p-value, the larger the node, the greater its statistical significance. The lines (edges) connecting the nodes in the graph represent the relationships between the terms. The open arrow heads indicate an ‘is_a’ relation between the GO terms, the solid arrow heads represent ‘part_of’ relations.

**
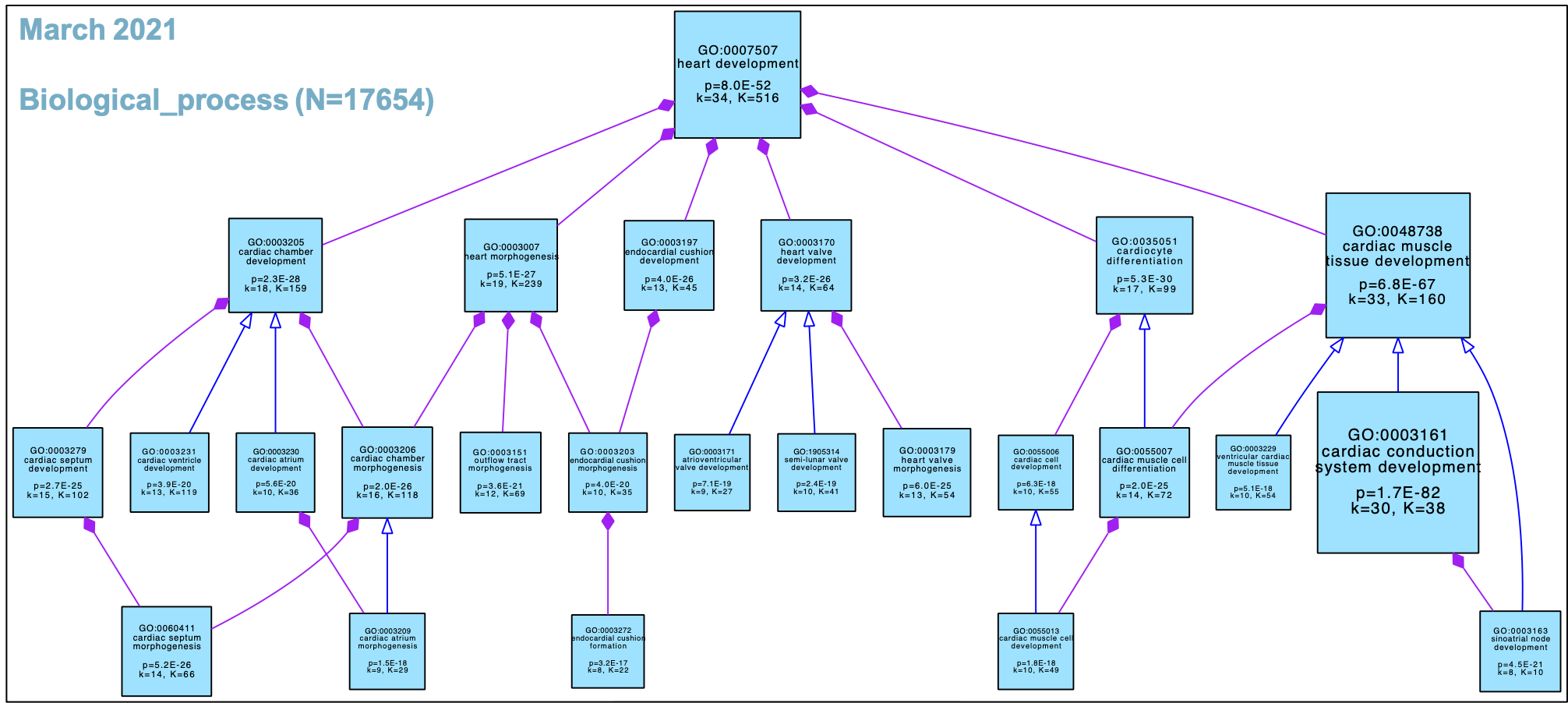
**

**
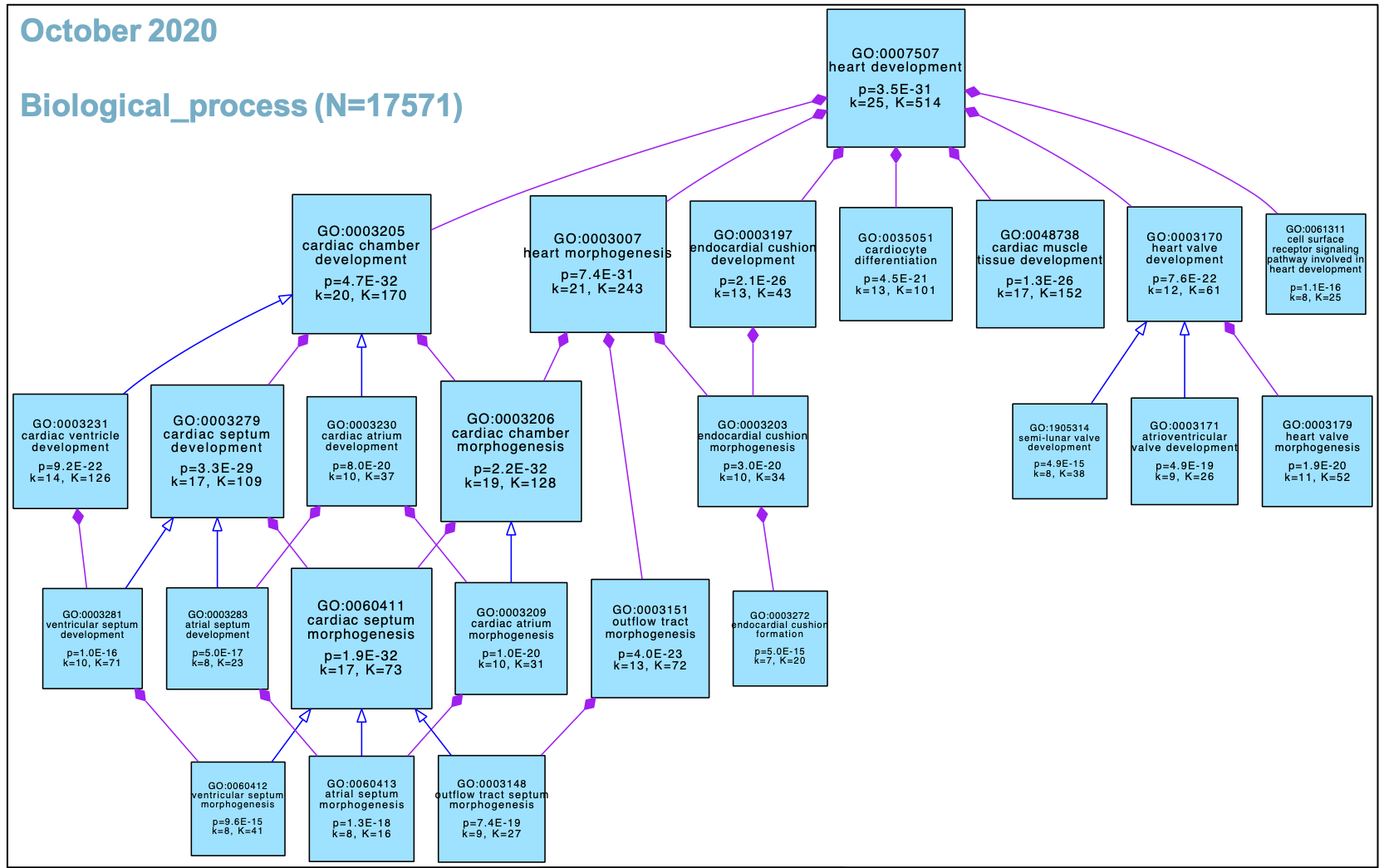
**
